# Prevalence of needle stick injury and its associated factors among nurses working in public hospitals of Dessie town, Northeast Ethiopia, 2016

**DOI:** 10.1186/s13104-018-3529-9

**Published:** 2018-06-28

**Authors:** Awoke Kebede, Hadgu Gerensea

**Affiliations:** School of Nursing, College of Health Sciences and Referral Hospital, AKsum University, Axum, Ethiopia

**Keywords:** Prevalence, Associated factors, Needle stick injury, Nurse

## Abstract

**Objective:**

Nurses are exposed to dangerous and deadly blood borne pathogens through contaminated needle stick injuries. This study was designed to assess prevalence of needle stick injury and its associated factors among nurses working in hospitals. Institution-based cross-sectional study design was used among 258 randomly selected nurses. Collected data was entered into Epi-Data version 3.1 and transferred to SPSS Version 20.0 for analysis. The degree of variables were assessed using adjusted odds ratio and its 95% confidence interval with *P* value (< 0.05).

**Results:**

Eighty-nine (34.5%) nurses self-reported receiving a needle stick injury in the previous 12 months. Work experience, working hour, personal protective, infection prevention guide line utilization and infection prevention training were significantly associated to needle stick injury.

**Conclusions:**

The needle stick injury in this study area was prevalent. The contributing factors to the injury were duration of working hours, experience, use of personal protective equipment and training.

## Introduction

The National Institute for Occupational Safety and Health (NIOSH) USA defines a needle stick injury as injuries that are caused by objects such as hypodermic needles, blood collection needles, cannula and needles used to connect parts of IV delivery systems. Health Professionals who are exposed to needles in their clinical activities are at high risk of acquiring a needle stick which may lead to a serious or fatal infection with blood borne pathogens such as hepatitis B virus (HBV), hepatitis C virus (HCV) or human immunodeficiency virus (HIV) [1, 2].

Approximately 3 million HCWs are exposed to blood borne viruses each year. Blood has been implicated as the source of the exposure in nearly all occupationally acquired infections. Exposures occur through needle sticks contaminated with an infected patient’s blood or through contact of the eye, nose or mouth with the patient’s blood [3–5]. On average, a healthcare worker (HCWs) in Africa suffers two to four needle stick injuries per year [6, 7].

NSI is one of the greatest risks faced by the front line health care worker. Yet, these exposures have often been considered as part of the job [8, 9]. Nurses are an integral part of clinical services and have primary responsibility for a greater proportion of patient care in most health care settings [10]. Nurses are at high risk to occupational hazards and injuries in the course of their day to day activities in the health care environment [11]. By considering the nature of the nurses’ working environment, responsibilities and duties, nurses are at high risk of numerous occupational hazards such as infectious disease, chemical risks, environmental risks, and psychosocial risks [12–15].

A few studies reported high prevalence of needle stick injury in central and eastern part of the country [16]. Therefore this study aims to assess the prevalence and associated factors among nurses working in public hospitals of Dessie town, Northeast Ethiopia.

## Main text

### Methodology

#### Study area and period

The study was conducted at public hospitals of Dessie town, Dessie, which is located at 401 km Northeast of Addis Ababa. The health facilities which are found in Dessie are two governmental hospitals, eight health centers, two NGOs clinics, three private hospitals 27 different size private clinics. The study was conducted from November 2015 to June, 2016.

#### Study design

Institutional based cross-sectional study design was conducted.

#### Sample size

A total of 313 samples were calculated using a single population proportion formula by assuming 5% marginal error and 95% confidence interval (σ = 0.05) and Prevalence of the needle stick injury 18.7% [17] and by adding 10% of non-response rate.

#### Sampling technique and procedures

About 258 subjects were selected using simple random sampling technique. The sample size had been distributed into each hospital according to proportional to population allocation. To select individual participant from each hospital, lottery method was used.

#### Data collection tools and techniques

Data was collected by using structured self-administered questionnaire adapted from previous similar literatures. Two days training was given to all data collectors and supervisors prior to pretesting. Eight data collectors who had completed diploma in nurses were recruited.

#### Data processing and analysis

The collected data was coded, cleaned and entered into Epi-Data version 3.1 and transferred to Statistical Package for Social Sciences (SPSS) version 20.0 for analysis. Bi-variable and multivariate logistic regression model were done to identify the relative importance of each predictor to the dependent variable by controlling for the effect of other variables. Those variables which were potential independent predictors on Bivariable analysis with P-value < 0.25 was entered to multivariable logistic regression analysis.

#### Ethical considerations

The Ethical approval was approved by the Institutional Review Board (IRB) of College of Health Sciences, Aksum University. Communications with the health center administrations was made through a formal letter obtained from Aksum University, College of Heath Science and TRHB. The objective and importance of the study was explained to the study participants. Data was collected after full informed written consent was obtained from participants aged 18 years and more, but age less than 18 years from the guardian.

### Results

#### Socio-demographic Characteristics

Two hundred fifty eight nurses were included in this study. The response rate was 97.6% (n = 252). The majority of the respondents 141 (56%) were females. More than two-thirds [171 (67.9%)] of the respondents reported being between the age of 20–29 years. One hundred and sixty eight (66.7%) respondents reported 168 (66.7%) having less than or equal to 5 years work experience. Additionally, 152 (60.3%) of participants had a degree and above. Two hundred one (79.8%) of the nurses work less than or equals to 40 h per week (See Table 1).

**Table 1:** Socio-demographic characteristics of nurses working in public hospitals of Dessie Town, March, 2016 (n = 252)

Variables	Description	Frequency	Percent (%)
Sex	Male	111	44
	Female	141	56
Age group	20–29 years	171	67.9
	30–39 years	35	13.9
	40–49 years	46	18.2
Experience	≤ 5 years	168	66.7
	5–10 yeas	31	12.3
	> 10 years	53	21.0
Educational status	Diploma	100	39.7
	Degree and above	152	60.3
Working hours per week	≤ 40 h	201	79.8
	> 40 h	51	20.2

#### Prevalence and circumstances of needle stick injury

The prevalence of needle stick injury among nurses working in public hospitals of Dessie town was 34.5% (n = 87) and 48.8% (n = 123) for the last year and throughout their career, respectively. From the total of respondents who had ever experienced needle stick injury, 84 (68.3%) were exposed to NSI only once. Hallow bore needle 71 (57.7%) followed by Suturing needle 35 (28.5%) was the most common devices involved in the injuries. Among injured nurses, 53.7% of the injuries occurred during night shift. The highest risky areas was surgical ward which accounts 26 (21.1%). One hundred three (82.7%) of accidents occurred in a noisy, dimly lit working environment. Regarding the degree of the injury, 69 (56.1%) respondents reported that the injury they were suffered was superficial. More than two-third 86 (66.9%) of injured nurses didn’t receive post exposure prophylaxis. Similarly, the majority of nurses 208 (82.5%) used personal protective equipment during a procedure while 123 (59.4%) reported using single glove as their personal protective equipment. Two hundred forty (95.2%) of nurses disposed of the needle using a safety box after completion of the procedures. only 177 (70.2) of nurses had knowledge of an infection prevention guideline and only three-quarters (n = 190) followed the guidelines. More than two-thirds (n = 177) and an almost equal number (n = 185) of nurses had received the hepatitis B vaccine and training on infection prevention respectively (See Table 2).

**Table 2:** Prevalence and circumstances of needle stick injury among nurses working in public hospitals of Dessie Town, March, 2016 (n = 252)

Variables	Description	Frequency	Percent (%)
NSI	Yes	123	48.5
	No	129	50.5
Duration	Within 1 year	87	34.5
	Before a year	36	14.3
How often	Once	84	68.3
	Two to four times	29	23.6
	≥ five times	7	5.7
	Didn’t recall	3	2.4
Materials leading to injury	Hallow bore needle	71	57.7
	Suturing needle	35	28.5
	Cannula	18	13.8
Working shift	Day	57	46.3
	Night	66	53.7
Working area/unit	OPD	10	8.1
	Emergency	19	15.4
	Pediatric ward	21	17.1
	Medical	19	15.4
	Surgical	26	21.1
	OR	15	12.2
	OBY-GYNI	7	5.7
	ICU	6	4.9
Condition of working environment	Bright light	16	13.0
	Dim light	47	38.2
	Noisy	56	45.5
	Room temperature	4	3.3
Injury type	Superficial	69	56.1
	Moderate	50	40.6
	Deep	4	3.3
Did you get Rx after injury?	Yes	37	30.1
	No	86	69.9
Did you use PPE?	Yes	208	82.5
	No	44	17.5
Type of PPE used	Single glove	123	59.4
	Double glove	73	35.3
	Mask	9	4.3
	Goggle	2	1.0
Did you safety box?	Yes	240	95.2
	No	12	4.8
Did your hospital has IP guideline?	Yes	177	70.2
	No	75	29.8
Did you follow IP guide line?	Yes	190	75.4
	No	62	24.6
Did you received HBV Vaccine?	Yes	177	70.2
	No	75	29.8
Did you received HBV IP training?	Yes	185	73.4
	No	67	26.6

Procedures played a role in the causes needle stick injury, injection is the leading one (24.4%) followed by suturing (16.3%).

#### Factors associated to needle stick injury

Based on a multiple logistic regression result, nurses with work experience greater than 10 years were more than six times at higher risk to sustain needle stick injury than those who work experience were less than or equals to 5 years with (AOR = 6.321, 95% CI 2.865–13.948). Nurses who worked greater than 40 h per were nearly three times at higher risk to sustain needle stick injury than those who worked hours less than or equals to 40 h per week with (AOR = 2.903, 95% CI 1.297–6.498). Nurses who do not use personal protective equipment during procedure were five times at more risk to sustain needle stick injury than their counter parts (AOR = 5.055, 95% CI 2.015–12.688). The risk of needle stick injury was nearly five times higher in nurses who didn’t follow infection prevention guidelines than those followed it (AOR = 4.623, 95% CI 2.052–10.416). Nurses who didn’t receive infection prevention training were nearly six times at more risk than nurses who received training (AOR = 5.780, 95% CI 2.691–12.415) (See Table 3).

**Table 3:** Factors Associated with Occurrence of Needle Stick Injuries among Nurses Working in Public Hospitals of Dessie Town, March, 2016 (n = 252)

Variables	Injury status		COR With 95% CI	AOR With 95% CI
	Yes	No		
Age group				–
20–29 years	71	100	1.00	
30–39 years	22	13	2.38 (1.126–5.046)*	
40+ years	30	16	2.641 (1.340–5.206)*	
Experience category				
≤ 5 years	73	95	1.00	1.000
5–10 years	15	16	1.220 (0.566–5.046)*	2.331 (0.925–5.871)**
> 10 years	35	18	2.530 (1.1327–4.842)*	6.321 (2.865–13.948)**
Working hours per week				
≤ 40 h	87	114	1.00	1.000
> 40 h	36	15	3.15 (1.619–6.108)*	2.903 (1.297–6.498)**
Use of PPE				
Yes	87	121	1.00	1.000
No	36	8	6.26 (2.773–14.127)*	5.055 (2.015–12.688)**
Use of safety box				–
Yes	113	127	1.00	
No	10	2	5.62 (1.206–26.191)*	
Availability of guide line				–
Yes	65	112	1.00	
No	58	17	5.88 (3.156–10.939)*	
Follow IP guide line				
Yes	73	117	1.00	1.000
No	50	12	6.68 (3.334–13.375)*	4.623 (2.052–10.416)**
Receiving IP training				
Yes	71	114	1.00	1.000
No	52	15	5.56 (2.916–10.623)*	5.780 (2.691–12.415)**

* Significantly associated at COR

** Significantly associated at AOR

### Discussion

Needle stick injuries are the most common route by which blood-borne viruses and/or infections such as HIV and hepatitis B and C. Such infections serve as high occupational risks and threats to healthcare workers, especially where basic rules of occupational safety and health are not implemented [18]. Among nurses who are working in public hospitals of Dessie town, the prevalence of needle stick injury in the previous 12 months prior to the survey was 34.5%. This finding is in line with results from previous study of Jigjiga (32.7%) and Hawassa (35.8%), Ethiopia [19, 20].

But this finding is higher than findings of Australia and Malaysia which is 17.7 and 27.9% respectively [21, 22] and lower than findings of Korea (70.4%) and Iran (54%) [23, 24]. Current result is also lower than studies of Addis Ababa and Jimma, Ethiopia (51.6 and 39.3%) respectively [25, 26]. The possible reason for difference in the proportion of injury could be the socio-demographic/economic status and cultural differences in self-reporting behavior. It also could be due to the difference in the study health facility setups, implementation of universal precaution, mixing up of study participants (all types of health care personnel Vs nurses only) and even the year of the study.

With regard to experience, the odds of sustaining needle stick injury was higher for nurses with work experience greater than 10 years than those whose work experience is less than or equals to 5 years with. This is in line with report from Portugal and Pakistan indicating experience greater than 10 years increases risk for sustaining needle stick injury and those with experience greater than 5 years are at greater risk to sustain NSI respectively [27, 28]. This could be explained by more exposure due to longer duration of services and hence more NSI among more experienced nurses as compare to those working for greater than 5 years. Younger workers apply more recently acquired knowledge into practice, while experienced familiarity may contribute to taking fewer precautions and paying less attention at work, which are likely to increase the chance of human error and contribute to risk behaviors.

Regarding to working hours per week, nurses whose working hour per week greater than 40 h were nearly three times at higher risk to sustain needle stick injury compared to those whose working hours per week is less than or equals to 40 h. This is similar with the finding of Iran and Sub-Saharan Africa [24, 29]. This is also in agreement with result from Jigjiga, eastern Ethiopia [19]. This could be explained by work load makes health professionals to be stressed, loss their ability to concentrate and fatigue, which are more likely to increase the chance of human error and contribute to a tendency towards risky behaviors and poor compliance with the precautions in general. It also suggests the problem of under-staffing in developing countries. Hence it has implications for policy makers and hospital administrators to ensure that working hours do not exceed than those prescribed in legislation.

Nurses who do not use personal protective equipment during procedure were more than four times at more risk to sustain needle stick injury than their counter parts. This is in line with previous report from Hawassa, south Ethiopia and other, which was 86.4% of HCWs use PEP consistently and those who don’t use were significantly associated accidental needle stick injury [5, 20]. This can also be explained by those who follow universal precaution are believed to have a good knowledge, and attitude and applied to prevent injury.

Those nurses who had not attended any training on prevention and management of needle stick injuries in their workplace were at a significantly greater risk of sustaining such injury compared with those who had attended some kind of training. This finding is similar with reports of Sub-Saharan Africa and Kenya [29, 30]. This also in line with finding in Jimma, south west Ethiopia [26]. This may be explained by effective training enhances awareness and improves skills of nurses to reduce unsafe behaviors and implementing organizational strategies to prevent exposure to reduce the risk of such injuries.

### Conclusions

This study revealed that more than one-third of the study participants had needle stick injury at least once in the previous 12 months. Inadequate occupational health and safety measures were factors associated with needle stick injury. So that ministry of health and heath professionals associations should create awareness on health professionals on safety measures.

## Limitation

By considering nurses are prone for needle stick injury we focuses on those health professionals but all health professionals are at risk and further study is need and systemic review is mandatory to made generalization. Furthermore, the study does not show cause and effect relationship.

## Abbreviations

CI: confidence interval
AOR: Adjusted Odd Ratio
SPSS: Statistical Package for Social Sciences
NSI: needle stick injury among nurses working in public hospitals of Dessie town
