# Expression of Concern to: Prevalence of needle stick injury and its associated factors among nurses working in public hospitals of Dessie town, Northeast Ethiopia, 2016

**DOI:** 10.1186/s13104-021-05480-4

**Published:** 2021-02-18

**Authors:** Awoke Kebede, Hadgu Gerensea

**Affiliations:** School of Nursing, College of Health Sciences and Referral Hospital, AKsum University, Axum, Ethiopia

## Expression of Concern: BMC Res Notes (2018) 11:413 https://doi.org/10.1186/s13104-018-3529-9

The Editor wishes to issue an expression of concern for this article [1].

After publication of this article it was brought to the Editor's attention that:

- Contrary to the statement included in the article, ethics approval for the study was provided by Mekelle University, not Aksum University
- Contrary to the statement included in the article, the study was conducted between February 2016 and November 2016
- Contrary to the statement included in the article, the data used and analyzed during the current study are not available
- The authors have not been able to provide redacted consent forms to confirm that informed consent was obtained from the study participants.

Neither of the authors provided a statement on whether they agree to this Expression of Concern or otherwise.
